# Single 5-nm quantum dot detection via microtoroid optical resonator photothermal microscopy

**DOI:** 10.1038/s41377-024-01536-9

**Published:** 2024-08-19

**Authors:** Shuang Hao, Sartanee Suebka, Judith Su

**Affiliations:** 1 Wyant College of Optical Sciences, University of Arizona, Tucson, AZ 85721 USA; 2 Wyant College of Optical Sciences and Department of Biomedical Engineering, University of Arizona, Tucson, AZ 85721 USA

**Keywords:** Imaging and sensing, Microscopy

## Abstract

Label-free detection techniques for single particles and molecules play an important role in basic science, disease diagnostics, and nanomaterial investigations. While fluorescence-based methods are tools for single molecule detection and imaging, they are limited by available molecular probes and photoblinking and photobleaching. Photothermal microscopy has emerged as a label-free imaging technique capable of detecting individual nanoabsorbers with high sensitivity. Whispering gallery mode (WGM) microresonators can confine light in a small volume for enhanced light-matter interaction and thus are a promising ultra-sensitive photothermal microscopy platform. Previously, microtoroid optical resonators were combined with photothermal microscopy to detect 250 nm long gold nanorods and 100 nm long polymers. Here, we combine microtoroids with photothermal microscopy to spatially detect single 5 nm diameter quantum dots (QDs) with a signal-to-noise ratio exceeding 10^4^. Photothermal images were generated by point-by-point scanning of the pump laser. Single particle detection was confirmed for 18 nm QDs by high sensitivity fluorescence imaging and for 5 nm QDs via comparison with theory. Our system demonstrates the capability to detect a minimum heat dissipation of 0.75 pW. To achieve this, we integrated our microtoroid based photothermal microscopy setup with a low amplitude modulated pump laser and utilized the proportional-integral-derivative controller output as the photothermal signal source to reduce noise and enhance signal stability. The heat dissipation of these QDs is below that from single dye molecules. We anticipate that our work will have application in a wide variety of fields, including the biological sciences, nanotechnology, materials science, chemistry, and medicine.

## Abstract (web summary)

We demonstrate ultra-sensitive photothermal microscopy with whispering gallery mode microtoroid resonators, detecting single 5 nm quantum dots with SNR > 10^4^ and achieving a minimum heat dissipation detection of 0.75 pW, which is below that from single dye molecules.

## Graphical abstract

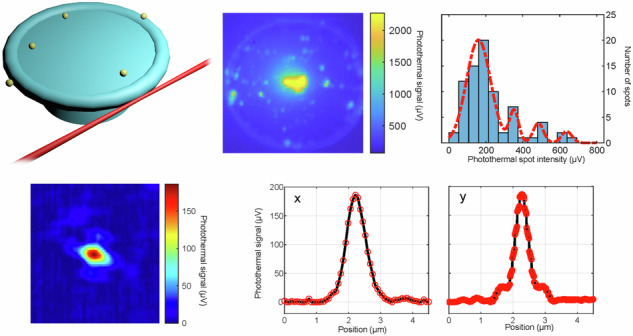

## Introduction

The detection of individual particles and molecules has had a significant impact in understanding protein dynamics^1^, DNA^2^ and RNA^3,4^ analysis, cellular imaging, nanotechnology and nanomaterials, and biomedical diagnostics, among other fields. Traditional single-molecule fluorescence-based detection methods such as Stochastic Optical Reconstruction Microscopy (STORM) or Photo-activated localization microscopy (PALM) are powerful tools to study molecular processes. Such techniques are widely used and valued for their low background noise and high sensitivity^5–7^. Fluorescence techniques, however, face limitations as they are restricted to a narrow range of molecular probes with high fluorescence quantum yields. Additionally, issues such as photoblinking^8–10^ and photobleaching^9^ limit their effectiveness.

As such, photothermal microscopy has emerged as a label-free non-invasive imaging technique. Photothermal microscopy measures localized variations in the refractive index of a sample’s surroundings. These variations result from the absorption of light by sample components, which in turn induce temperature changes in the surrounding region^11^. Photothermal microscopy can provide insight into the optical and thermal properties of materials^12–15^ and biological structures^16–18^. Its high sensitivity renders it advantageous across a diverse array of applications, including material science^11,19^, nanotechnology^20–22^, biological imaging^23,24^, and thermal metrology^25–27^.

Currently, photothermal heterodyne imaging (PHI)^28^ is the predominant method for photothermal microscopy of single nano-objects. PHI can detect gold nanoparticles as small as 1.4 nm in diameter^28^ with a signal-to-noise ratio (SNR) over 10 and measure the absorption spectra of single ~7 nm quantum dots (QDs) at room temperature^29^ with an SNR < 10. By using multiple pump beams, PHI can perform multiplexed imaging, enabling simultaneous targeting and detection of gold and silver particles^30^ or dynamic imaging of mitochondria and lysosomes in living cells^17^. Nanomechanical silicon nitride drums have also been used as mechanical transducers for photothermal imaging of single dye molecules, specifically Atto 633, with a heat dissipation of 6.3 pW^31^. Although photothermal microscopy coupled with mechanical transducers exhibits high sensitivity, it is limited to operating under high-vacuum conditions.

Here, to perform ultra-sensitive photothermal imaging in ambient air at room temperature, we use whispering gallery mode (WGM) microtoroid resonators as detectors in photothermal microscopy and achieve single 5 nm QDs detection with an SNR over 10^4^ and with a simpler system and alignment requirement than PHI. WGM microtoroid optical resonators can measure small temperature changes induced from the heat dissipation of molecules. They are a class of optical microcavities known for their ultra-high quality (Q) factors, making them suited for a diverse set of applications, including single molecule detection^32–34^, biochemical detection^35–38^, and frequency comb generation^39–42^. The Q-factor characterizes the efficiency and energy loss within the resonator and is mathematically expressed as $Q={\lambda }_{0}/\varDelta \lambda$, where *λ*_0_ is the WGM resonant wavelength and *Δλ* is the full width at half maximum linewidth (FWHM) of the WGM. The ultra-high Q factor of WGM resonators results in a small mode volume^43–45^, which in turn greatly enhances light-matter interaction. In addition, the narrow resonances of WGM enable precise measurement of the resonance shift. These high Q factors can enable photothermal microscopy with high sensitivity and precision.

Among various types of WGM optical resonators, such as microspheres^46–48^, microdisks^49–51^, microbubbles^52^ and microtoroids^53–55^, microtoroids stand out due to Q-factors^56^ in excess of ${10}^{8}$, and a flat disk plate that facilitates the placement of nanoabsorbers. Previously, photothermal spectroscopy of single polymers with absorption cross-section of 6.7 ${{\rm{nm}}}^{2}$ was demonstrated by measuring the resonance shift of microtoroids^57^; however, detecting smaller particles has proven challenging^11^ due to the high background signal generated from the microtoroid’s silicon pillar. To overcome this limitation, all-glass microtoroids^58^ have been designed and fabricated to minimize unwanted absorption from the supporting pillar. The all-glass fabrication process, however, leads to a sacrifice in the Q-factor, resulting in values around ${10}^{6}$.

To preserve the high Q-factor of the microtoroid, a re-etching process can be applied to decrease the size of the supporting pillar. This adjustment has been proven to significantly enhance sensitivity without compromising the microtoroid’s high Q-factor^59^. The re-etching process of the microtoroid significantly decreases the size of the microtoroid pillar, providing two benefits: (1) it reduces the area with high background signal and (2) provides better thermal isolation of the microtoroid, enhancing the photothermal effect. Here, we demonstrate photothermal microscopy based on a re-etched microtoroid and low amplitude modulation (AM) frequency of the pump laser. We use the proportional-integral-derivative (PID) controller output signal to measure the resonance shift instead of the error signal as was previously used^27^. This is because the re-etched microtoroid has enhanced photothermal sensitivity with a lower cut-off frequency^59^. The lower cut-off frequency limits the AM frequency, which increases the background noise of the photothermal signal.

In previous work, the resonance shift from the error signal was measured, but because the AM frequency is too high, the probe laser wavelength doesn’t tightly follow the resonance shift. By using a low AM frequency and employing the PID output instead of the error signal output, we can more closely track the resonance shift. This refinement allows us to achieve better sensitivity and performance without suffering from increased noise due to the decreased AM frequency.

In our approach, nanoparticles are deposited onto the top surface of the microtoroid. Upon illumination with a pump light at a wavelength of 405 nm, absorption of light by the nanoparticles leads to localized heating and dissipation within the resonator. In the case of the fused silica microtoroid, it possesses a positive thermal expansion coefficient ($5.5\times {10}^{-7}\,{{\rm{K}}}^{-1}$)^60^ and a positive thermo-optic coefficient ($8.6\times {10}^{-6}\,{{\rm{K}}}^{-1}$)^61^. Both positive coefficients contribute to the optical path length increasing, consequently leading to a redshift in resonance wavelength. The photothermal signal is directly related to the heat dissipation from the optical absorbing particles. The shift in the WGM resonance is proportional to the absorption cross-section of the nanoparticles. To detect these resonance shifts, we utilize an additional probe laser with a wavelength of 780 nm, which is intentionally detuned far away from the pump laser to avoid interference from nanoparticle or molecule absorption. Compared to the high frequency amplitude modulation of the pump laser used by others, we use low frequency modulation of the pump laser to enable the frequency lock-in system to track the oscillating resonance shift signal closely and accurately, improving the SNR of the photothermal signal.

Previously, we developed a system called Frequency Locked Optical Whispering Evanescent Resonator (FLOWER)^62–67^, which combines optical microcavities with frequency locking and data processing to enable the detection of single macromolecules. Unlike conventional methods that involve scanning the wavelength of a tunable probe laser, where the measuring time is constrained by the laser scanning speed and resonance measurement accuracy is compromised by laser drift, FLOWER uses frequency locking^68^ to reduce the response time and enhance the accuracy of resonance shift measurements.

**Fig. 1:**
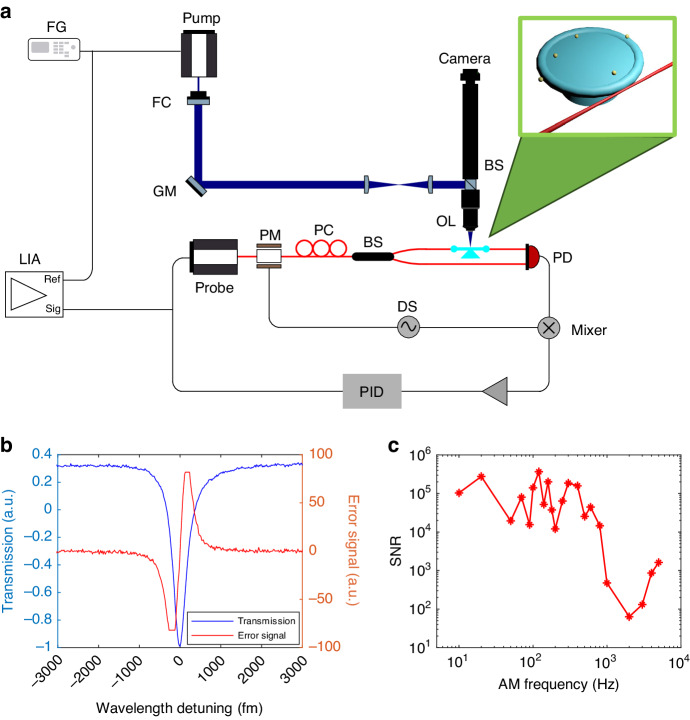
Photothermal microscopy based on microtoroid. **a** Photothermal microscopy system setup. The top right is the particle placed microtoroid coupling to tapered fiber. Key components include FG Function Generator, FC Fiber Collimator, GM Galvo Mirror, LIA Lock-in Amplifier, PM Phase Modulator, PC Polarization Controller, BS fiber Beam Splitter, OL Objective Lens, PD Photodetector, DS Dither Signal, PID Proportional-Integral-Derivative controller, Probe laser and Pump laser. **b** The resonance transmission of the microtoroid (blue curve) is acquired by the probe laser scanning. The Q factor of the resonance is $1.86\times {10}^{6}$. The error signal (red curve) equals zero at the resonance peak wavelength. **c** The SNR of the photothermal signal of DiagNano (DN) 800 QDs (size:5 ~ 6 nm). The time constant is set as 1 s in the lock-in amplifier

Our experimental setup integrates FLOWER with photothermal microscopy. The experimental setup shown in Fig. 1a involves coupling a probe laser with a wavelength of 780 nm into the microtoroid resonator through a tapered optical fiber. To optimize the coupling efficiency between the tapered fiber and microtoroid, we control the polarization of the probe laser using a polarization controller (PC). A 25 MHz oscillation dither signal (DS) is applied to drive the phase modulator, resulting in phase modulation of the probe laser. Subsequently, the phase-modulated probe laser is split into two arms through a fiber-coupled beam splitter (BS): one arm carries the signal light coupled into the microtoroid, while the other one serves as the reference light. Both the signal and reference light are then received by a high-bandwidth balanced photodetector (PD). This balanced detector plays a crucial role in removing common noise from the probe laser. By multiplying the balanced photodiode’s electrical output signal by the dither signal and time-averaging, we obtain an error signal that is proportional to the wavelength detuning between the probe laser and the WGM resonance of the microtoroid. Figure 1b shows the resonance transmission spectra and its corresponding error signal, where the error signal becomes zero at the resonance when the probe laser perfectly matches the microcavity WGM resonance. Utilizing this error signal in a feedback loop with the tunable laser controller, the PID controller minimizes the error signal, thereby ensuring accurate and stable frequency locking of the probe laser to the WGM resonance.

**Fig. 2:**
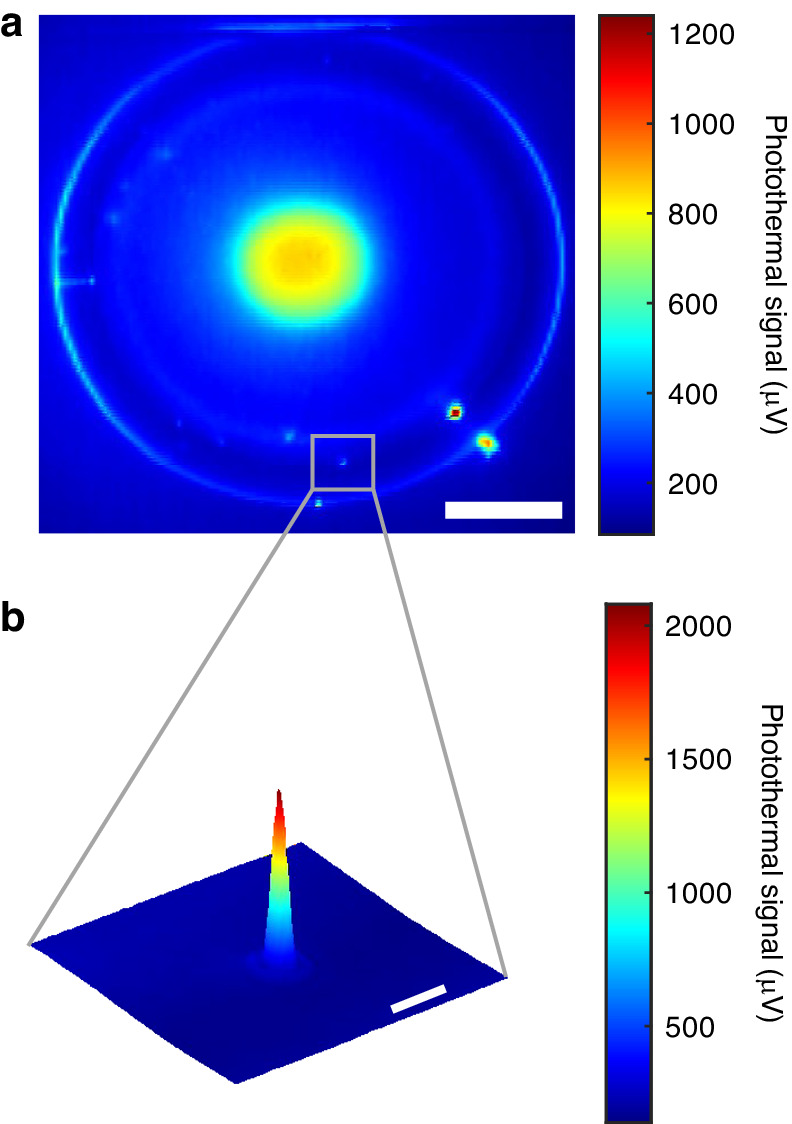
Photothermal map of the microtoroid with Au nanosphere binding. **a** Coarse photothermal map of the whole microtoroid. The diameter of Au nanosphere on the microtoroid is 100 nm. Scale bar, 20 µm. **b** Fine photothermal map of single 100 nm Au nanospheres marked in (**a**). Scale bar, 2 µm. The 2D photothermal maps are generated by scanning the selected area line-by- line using the pump laser

In the PID control system, the output of the PID controller represents the resonance shift signal. Conversely, the error signal reflects the wavelength detuning between the probe laser and the WGM resonance. The oscillatory resonance shift with low frequency can be tracked more effectively by the PID controller output signal instead of the error signal. The PID method boasts an SNR exceeding tenfold that of the error signal method (Supplementary Information Section 1). During photothermal imaging experiments involving nanoparticles, we deposit QDs or Au nanospheres onto the microtoroid. However, the introduction of these nanoparticles induces additional losses, leading to a reduction in the Q-factor of the microtoroid. In Fig. 2a, the Q-factor of the microtoroid with Au nanospheres is measured to be $1.86\times {10}^{6}$. The selection of this specific Q-factor resonance is guided by careful consideration of its impact on the photothermal signal detection range. While higher Q factors generally lead to increased sensitivity, they may limit the effective tracking range of the resonance shift and risk destabilizing the frequency locking due to high photothermal signals. The Q-factor values achieved in the ${10}^{6}$ range are comparable to those observed in single-molecule detection using WGM resonators^69–71^. Choosing a resonance with a Q factor in this range strikes a balance, enabling imaging of the entire microtoroid without saturation distortion while preserving good sensitivity. In future experiments aimed at detecting small single molecules with low absorption, selecting a resonance with a higher Q factor can further enhance sensitivity.

In our photothermal microscopy system (Fig. 1a), an additional continuous wave (CW) laser emitting at 405 nm serves as the pump laser. This specific wavelength resides at the boundary between visible and UV light spectra, aligning with the characteristic absorptions exhibited by a wide range of molecules and particles. This wavelength choice guarantees the generation of a robust photothermal signal. Significantly, the pump laser’s wavelength is detuned from that of the probe laser, preventing any potential interference between the two.

Photothermal microscopy based on the WGM microcavity can be constructed using separated pump and probe light sources. The pump light travels through free space, guided by a galvo mirror (GM) that controls the position of the laser spot on the microtoroid^59^. The probe light, on the other hand, is coupled to the microtoroid through a tapered fiber. The pump laser output is initially directed through a fiber collimator (FC) to convert it into a free-space collimated beam. This collimated beam is then transmitted through a GM scan system and focused on the top surface of the microtoroid using a 60X objective lens. A relay lens, which is comprised of a scan lens and a tube lens, is positioned between the GM and the cubic beam splitter. The GM scan system controls the position of the laser spot on the microtoroid plane by adjusting the incident angle of the pump beam. By employing a two-axis rotation for the GM, a 2D spatial scan of the pump laser on the microtoroid can be achieved. The pump laser is amplitude modulated with a 203.7 Hz oscillating signal from a function generator. The resulting 203.7 Hz AM signal in the resonance shift is detected by FLOWER. The amplitude of this oscillation directly correlates with the heat dissipation caused by the pump laser beam, effectively representing the photothermal signal. Figure 1c depicts the SNR of the photothermal signal of single 5-6 nm QD as a function of the AM frequency. Previous research has demonstrated that microtoroids with small pillars exhibit a limited bandwidth of 400 Hz for AM in the photothermal heating signal^59^. The small pillar of the microtoroid causes a high photothermal sensitivity but sacrifices response speed. In our study, the microtoroids display a cut-off photothermal frequency at 300 Hz, as depicted in Fig. 1c. To capitalize on the high SNR and achieve accurate real-time tracking of the photothermal signal, we utilize a lock-in amplifier operating at the AM frequency of 203.7 Hz (Additional details regarding the AM frequency selection are provided in Supplementary Information Section 1). This combination of techniques significantly improves the SNR of the image acquired by the photothermal microscopy based on the re-etched microtoroid optical resonator.

## Results

### Imaging of Au nanospheres

The use of Au nanoparticles in conjunction with antibody labeling is a valuable technique for biomolecule detection^72^. Au nanoparticles can serve as markers or tags that can be easily visualized and detected due to their distinct optical properties. Therefore, a 100 nm Au nanosphere was first selected as a target particle. The intensity of the 405 nm pump laser spot is $17.4\,{\rm{KW}}\cdot ({{\rm{cm}}})^{-2}$, which is significantly below the melting point of the Au particles. A 2D scan of the microtoroid is first performed using the pump laser beam. The amplitude of the resonance shift is recorded with a double-locking mechanism, both from FLOWER and from the lock-in amplifier. The photothermal signal of each pixel during the 2D scan is the same and is longer than the time constant of the lock-in amplifier. The Au nanospheres are placed on the microtoroid using an aerosol generator. The 100 nm Au nanosphere physically binds to the microtoroid. Figure 2a presents the photothermal map of the entire microtoroid with Au nanospheres. The microtoroid structure consists of glass material for the disk and toroidal rim parts, which exhibit minimal absorption of the 405 nm pump laser. The supporting pillar of the microtoroid is made of silicon, which has high absorption. This configuration results in a low photothermal signal being generated by the microtoroid itself, In contrast, the pillar produces a strong and distinct photothermal signal. As a result, the effective detection area for particles is primarily the microtoroid area excluding the pillar. To expand the effective area for nanoparticle detection, secondary etching is employed during microtoroid fabrication, reducing the pillar size. Additionally, the smaller pillar contributes to the heat insulation of the microtoroid, thus improving the sensitivity of the photothermal signal. In Fig. 2a, multiple photothermal hotspots are distributed around the rim of the microtoroid, representing the presence of 100 nm Au nanospheres. The absorption cross section of 100 nm Au nanosphere is calculated as ${\sigma }_{{\rm{Au}}}=1.97\times {10}^{4}\,{{\rm{nm}}}^{2}$. The heat dissipation is ${P}_{{heat}}=3.43\times {10}^{6}\,{\rm{pW}}$. The calculation details are provided in Supplementary Information Section 2. A fine photothermal map of an individual 100 nm Au nanosphere is shown in Fig. 2b. This result highlights the ease with which the 100 nm Au nanosphere can be detected using photothermal microscopy. To further explore the detection limits of this photothermal microscopy system, smaller nanoparticles were then chosen as target particles.

### Imaging of QDs

**Fig. 3:**
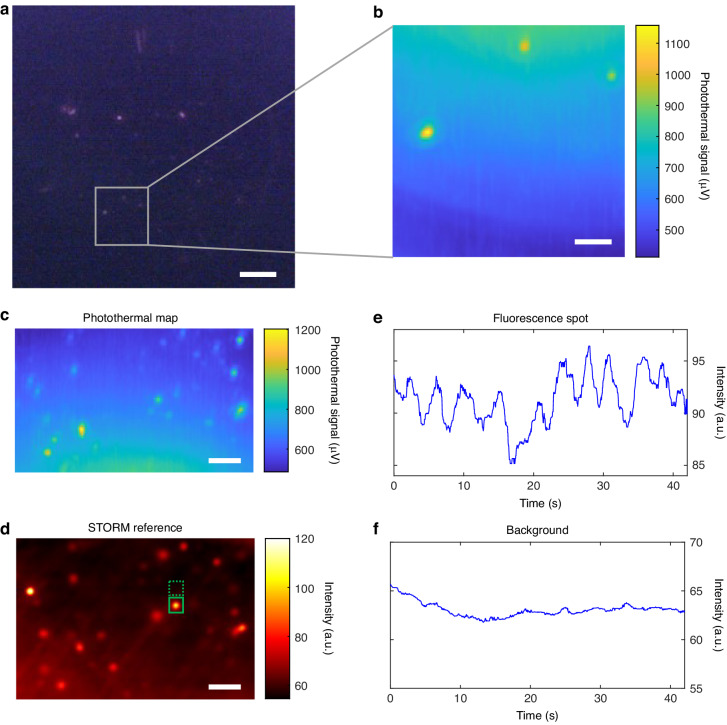
Photothermal image of Qdot 800 QDs (size:18 ~ 20 nm). **a** Fluorescence image of Qdot 800 QDs on the microtoroid. Scale bar, 10 µm. **b** Photothermal image of three individual Qdot 800 QDs in the gray square marked area in (**a**). Scale bar, 2 µm. **c** Fine photothermal map of the Qdot 800 QDs on another microtoroid. Scale bar, 3 µm. **d** The superimposed frames image illustrating the same area as shown in (**c**), acquired through high sensitivity fluorescence imaging. Scale bar:3 µm. **e** Intensity profile of the spot within the solid green square region in (**d**). **f** The background signal captured within the green dashed square region of (**d**)

To demonstrate the high sensitivity of photothermal microscopy for single nanoparticles, we specifically opted for Qdot 800 QDs (Thermo Fisher Scientific) as our target particles based on their well-established and recognized characteristics. Firstly, the QDs emit stable fluorescence light at a peak wavelength of 793 nm. The fluorescence image can serve as a reference image for the photothermal map. Secondly, unlike fluorescence dyes, QDs do not undergo photobleaching, enabling longer observation times and stable fluorescence images. Thirdly, Qdot 800 QDs exhibit strong absorption in the UV spectrum, which matches the 405 nm wavelength of the pump laser. The diameter of the Qdot 800 QDs ranges from 18 nm to 20 nm. Figure 3a displays the fluorescence image of Qdot 800 QDs on a microtoroid, covering the entire WGM resonator and serving as a reference for subsequent photothermal imaging. In Fig. 3b, three QDs are scanned using photothermal microscopy within the marked region indicated in Fig. 3a. The resulting photothermal image reveals three hot spots in the disk area of the microtoroid, corresponding to the three fluorescence spots observed in Fig. 3a. To confirm that the photothermal signals originate from single QDs, a fine photothermal map of Qdot 800 QDs on another microtoroid is presented in Fig. 3c. Additionally, the same microtoroid is imaged using high sensitivity fluorescence imaging, capturing the blinking behavior of the QDs. The superimposed frames of the video form the fluorescence image shown in Fig. 3d. The characteristic single step blinking behavior of single QDs is evident in Fig. 3e, while the background signal from the fluorescence video is depicted in Fig. 3f. Both the spot intensity and background signal are filtered by a median filter. The intensity of the single step blinking signal surpasses the background noise level, providing evidence for the presence of a single absorber and emitter. The sensitivity of the photothermal signal depends on the location of the absorber (Supplementary Information Section 4). The sensitivity near the equator of the microtoroid is higher than in the location near the pillar. Moreover, when confining the photothermal spot selection to the annular region between points B and C, as illustrated in Fig. S3a (see Supplementary Information Section 4), the variation in photothermal responsivity remains relatively consistent, with an ~20% margin of error at most. Photothermal microscopy enables precise identification of target locations on the microtoroid and measurement of the absorption cross-section.

**Fig. 4:**
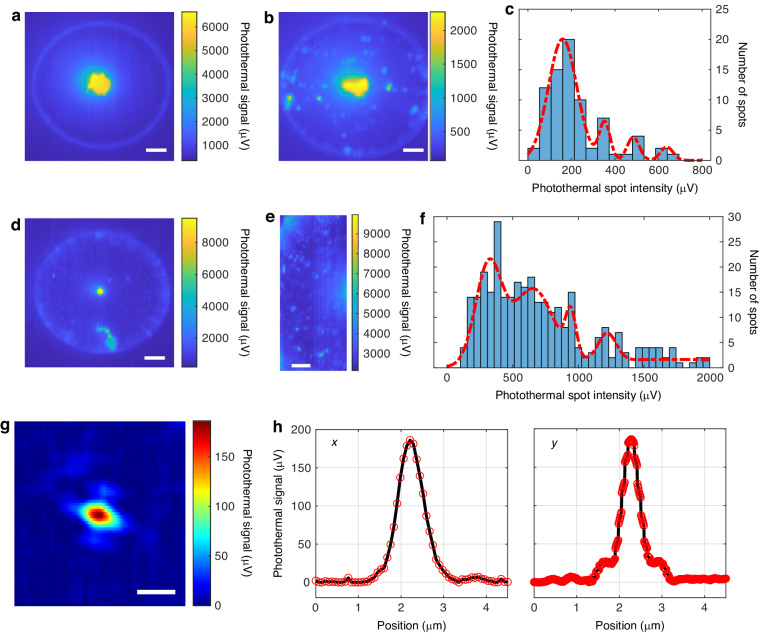
Photothermal map comparison of microtoroid with QDs. **a** Photothermal map of the microtoroid prepared in the same way, but without any QDs. Scale bar, 10 µm. **b** Photothermal map of microtoroid with DN 800 QDs (size:5-6 nm). Scale bar, 10 µm. **c** Histogram of spot maximum intensities for DN 800 QD in (**b**). **d** Photothermal map of microtoroid with a mixture of DN 800 QDs (size: 5–6 nm) and Qdot 800 QDs (size:18–20 nm). Scale bar, 10 µm. **e** Part of the microtoroid in d is photothermally imaged with high spatial resolution. Scale bar, 5 µm. **f** Histogram of spot maximum intensities for mixed QDs in (**d**). The red curves in (**c**) and (**f**) are GMM fitting. **g** Photothermal spot of a single DN 800 QD with background signal removed. Scale bar, 1 µm. **h** Profile cut through the photothermal peak of a single QD in both the x and y directions, as indicated in (**g**)

To explore the detection limits of photothermal microscopy, we employed smaller QDs, DiagNano (DN) 800 (CD Bioparticles) which are 5-6 nm in diameter. One concern was the possibility of chemical contamination on the microtoroid during the coating process, which could contribute to undesired photothermal signals. To address this, we performed a control experiment by handling the microtoroid with the same coating process but without introducing any QDs. The resulting photothermal map of the control group as shown in Fig. 4a, exhibited no detectable photothermal spots. This finding provided evidence that either there was no contamination on the microtoroid during the chemical coating process or, if present, the remaining chemical material had minimal absorption at 405 nm, thus contributing little to the photothermal signal. Next, we conducted photothermal imaging of DN 800 QDs on the microtoroid, (Fig. 4b). Although we attempted to observe the DN 800 QDs using fluorescence microscopy in combination with an EMCCD camera, their small absorption cross-section and lower quantum yield made it challenging to observe their fluorescence or photoblinking behavior of individual QDs. Here, we analyzed the fine photothermal map of the microtoroid (Fig. 4b) and constructed a histogram of the photothermal spot intensity (Fig. 4c). Notably, individual nanoparticles exhibit a monomodal distribution^12,73^, while the presence of both aggregated QDs and single QDs on the microtoroid resulted in multiple peaks. Each peak followed a Gaussian distribution, representing distinct orders of QD aggregate cluster sizes. The peak with the lowest mean intensity indicated individual QDs, while the peak value of higher-order aggregates was the product of the order number and the distribution peak value of an individual QD. This relationship facilitated the differentiation of different QD aggregates and individual QDs based on their photothermal spot intensity distribution. To perform the analysis, we employed a Gaussian Mixture Model (GMM) with the iterative Expectation-Maximization (EM) algorithm to fit the histogram (Fig. 4c). Our results revealed that the photothermal signal of a single DN 800 QD measured 160 ± 65 µV, representing 80.0% of the 77 spots. Furthermore, aggregates of two QDs generated a photothermal signal of 353 ± 25 µV (9.5% of the spots), aggregates consisting of three QDs exhibited a photothermal signal of 484 ± 28 µV (6.6% of the spots), and four QDs exhibited a photothermal signal of 638 ± 29 µV (3.9% of the spots). The photothermal spots primarily corresponding to individual QDs, constituted 80% of the total observed spots, while higher-order DN 800 QD aggregates showed diminishing proportions as the order number increased. Moreover, aggregates with more than four QDs were scarce on the microtoroid surface (Fig. 4b). The individual heat dissipation of DN800 QDs, as outlined in Table 1, is calculated to be 71.3 pW, corresponding to a photothermal signal of 159.6 µV in the histogram (Fig. 4c). This results in a photothermal sensitivity for heat dissipation of 2.24 µV/pW. Within the histogram, the minimum recorded photothermal spot intensity is 40.2 µV, indicating a heat dissipation of 17.9 pW. The noise floor, measured at 1.7 µV (Supplementary Information Section 5), establishes the minimum detectable heat dissipation in the photothermal map at 0.75 pW. It is noteworthy that detecting such small QDs using SEM would be difficult due to the presence of other contaminant particles that would make the QDs hard to find. In FLOWER based photothermal microscopy, such particles remain invisible due to their low absorption at 405 nm.

**Table 1:** Optical Properties of Qdot 800 and DN 800 QDs

	Quantum yield	Molar extinction coefficient (${{\bf{M}}}^{{\boldsymbol{-}}{\bf{1}}}{\boldsymbol{\bullet }}{{\bf{cm}}}^{{\boldsymbol{-}}{\bf{1}}}$)	Absorption cross section (${{\bf{nm}}}^{{\bf{2}}}$)	Fraction of heat dissipation	Absorbed Power (pW)	Heat dissipation (pW)
Qdot 800	62%	8.0 × ${10}^{6}$	3.06	68.3%	531.4	363.1
DN 800	16%	1.3 × ${10}^{6}$	0.49	92.8%	77.4	71.3

(Derivation details are provided in the Supplementary Information)

To further evaluate the discriminating capabilities of FLOWER based photothermal microscopy for different particles, we applied a mixture of DN 800 and Qdot 800 QDs to the microtoroid surface. The resulting photothermal image of the entire microtoroid is presented in Fig. 4d, with a finer photothermal image of the left part shown in Fig. 4e. Figure 4e exhibits photothermal spots with varying intensities, where strong photothermal spots correspond to Qdot 800 QDs and weaker spots represent DN 800 QDs. This is based on their cross-section absorption characteristics detailed in Table 1. To quantitatively analyze the photothermal spots, we constructed a histogram of photothermal spot intensities in Fig. 4f and employed the same fitting methodology to identify five Gaussian distribution peaks. We note that the fifth peak at 1696.5 µV is not clearly shown due to its large standard deviation. This is because the concentration of the larger QDs was kept low in this experiment. Photothermal spots from single Qdot 800 QDs exhibited an intensity of 1697 ± 794 µV, constituting 21.0% of the 327 spots. In comparison, the photothermal signal from single DN 800 QDs was measured to be 317 ± 100 µV, accounting for 31.3% of the spots. The photothermal signal of single DN 800 QDs differs between Fig. 4c and f, primarily due to sensitivity variations resulting from the microtoroid’s pillar size. The smaller supporting pillar in Fig. 4d provides better thermal isolation, contributing to enhanced photothermal sensitivity. Moreover, two DN 800 QD aggregates displayed a photothermal signal of 653 ± 153 µV (36.3% of the spots), while three DN 800 QD aggregates exhibited a photothermal signal of 943 ± 40 µV (5.4% of the spots). Additionally, four DN 800 QD aggregates demonstrated a photothermal signal of 1219 ± 69 µV (6.0% of the spots). Consequently, the photothermal signal ratio of Qdot 800 QDs to DN 800 QDs was ~5.4. This experimental photothermal signal ratio for single QDs closely corresponds to the theoretically calculated heat dissipation values detailed in Table 1, exhibiting a difference of 5.2%.

Employing this analysis of photothermal intensity, we selected a single DN 800 QD on the microtoroid for high-resolution scanning (x direction: 75 nm/pixel, y direction: 9.4 nm/pixel). The resulting photothermal image is presented in Fig. 4g, with both x and y directions indicated in Fig. 4h. Typically, after performing a high-resolution photothermal spot scan and adjusting the microtoroid position to match the pump laser’s focal point, photothermal peak intensities will increase compared to the fine photothermal map of multiple spots. In Fig. 4g, the photothermal peak’s value measures 186.3 ± 1.7 µV. For a single DN 800 QD (size: 5-6 nm), this yields a SNR of $1.2\times {10}^{4}$. Calculations based on the experimental setup information establish the heat dissipation of a single 5 nm DN 800 QD to be 71.3 pW. The high (>10^4^) SNR observed for a single 5 nm QD using our photothermal microscopy underscores our potential to detect single small molecules.

## Discussion

In summary, we demonstrated that FLOWER based photothermal microscopy using re-etched microtoroids can detect single nanoparticles, as small as 5 nm QDs, with a SNR exceeding 10^4^. The detection limits of photothermal microscopy are determined to be 0.75 pW in heat dissipation, significantly greater than the tens of pW noise floor reported previously^27^ and more than two orders of magnitude smaller than the 0.34 nW heat dissipation power reported for single dye molecules in photothermal detection^74^. This provides compelling evidence of our photothermal microscopy’s ability to detect single molecules. The significant advancement in detection limits is attributed to the enhancement in photothermal sensitivity. Despite the higher photothermal sensitivity of the toroid rim area, we deliberately selected the microtoroid disk area adjacent to the rim for detecting QDs and measured their absorption based on the photothermal signal. This choice was influenced by the Fano resonance spectra exhibited by nano-particles in the toroid rim area^27^, which could complicate their intrinsic absorption measurements. By choosing the area near the toroidal rim area, we maintained high photothermal sensitivity with minimal differences in measurement values, allowing accurate measurement of the nanoparticle’s absorption based on the photothermal signal. A 2D photothermal image was generated point-by-point through a galvo mirror scan. This enabled detailed visualization of the absorption properties and specific binding locations of the target nanoparticles on the microtoroid’s surface. Unlike fluorescence techniques, the photothermal signal comes from the heat dissipation of absorbed light, thereby enabling the detection of non-luminescent materials. Nonetheless, fluorescence images still served as a valuable reference for photothermal mapping. The detection of single-step quantum blinking behavior from fluorescence microscopy and the intensity distribution of photothermal spots further validated the identification of individual QDs rather than aggregates.

While we have achieved high sensitivity and discrimination capabilities in photothermal microscopy, there are opportunities for improvement. For instance, increasing the phase modulation frequency and adjusting the AM frequency accordingly could reduce the response time and acquisition time of a photothermal image. Moreover, future advancements may involve spectroscopy measurements by varying the pump laser’s wavelength or exciting with different wavelengths to enable multicolor imaging^30^. In future work, although not necessary for many applications, we can also combine this system with specific capture agents such as aptamers or sorbent polymers^35,75^ to provide enhanced specificity .The integration of photothermal microscopy with WGM resonators opens possibilities for real-time observation of dynamic changes and interactions of target molecules. We believe that overall, FLOWER based photothermal microscopy represents a versatile platform for label-free imaging and single-molecule detection. The demonstrated high sensitivity and discrimination capabilities pave the way for advancements in nanoscale imaging and characterization techniques.

## Materials and methods

### Fabrication of re-etched microtoroids

The fabrication process of microtoroid resonators has been previously described^53^. The microtoroid resonators are fabricated on silicon wafers with a 2 μm layer of thermally grown silica. First, circular disc patterns of photoresist with a diameter of 100 µm are created on the top silica layer of the silicon wafer. Solution containing 1:6 v/v at room temperature, which contains HF (7–15%). After wet etching, any residual photoresist and contaminants are removed using acetone and isopropyl alcohol (IPA). The wafer is then subjected to a post-bake at 130 °C to remove moisture^65^. The remaining silica disks act as etch masks during exposure to xenon difluoride (XeF_2_) gas, resulting in the uniform undercutting of the silica disks and the formation of silicon pillars that support the silica disks. A thermal reflow process using a CO_2_ laser is employed which provides a smooth surface finish and shapes the silica disk into a microtoroid. Subsequent re-etching of the microtoroid using XeF_2_ gas is performed to decrease the diameter of the supporting pillars to meet the requirements of the experiment.

### Au nanosphere binding to the microtoroid

A 100 nm Au nanosphere solution (nanoComposix) was diluted 100 times with HPLC-grade deionized water to achieve a concentration of 5 μg/mL. The diluted Au nanosphere solution was then injected into an aerosol generator. The aerosol generator includes a dryer that removes the liquid water from the Au nanosphere aerosol, resulting in dry aerosol particles. To perform the spraying process, the microtoroid chip was positioned ~1 cm below the aerosol output nozzle in a fume hood. This allowed the Au nanospheres to bind to the microtoroid's surface.

### Binding QDs to the microtoroid

After the fabrication of the re-etched microtoroid, the microtoroid chip was cleaned with ethanol and dried by nitrogen gas spray to remove any potential contamination. The microtoroid chip was then treated with a solution containing 2% v/v of 3-aminopropyl-triethoxysilane (APTES) and ethanol for amine functionalization. The chip was incubated in this solution for 2 min at room temperature. After incubation, the microtoroid chip was rinsed with fresh ethanol and IPA, followed by drying using a flow of nitrogen gas. Next, a mixed QD solution was prepared with 100 mM1-ethyl-3-(3-dimethylaminopropyl) carbodiimide (EDC) and 100 mM N-Hydroxysulfosuccinimide sodium salt (sulfo-NHS) in 0.1 M 2-(N-morpholino) ethanesulfonic acid (MES) buffer (pH = 6.7). The microtoroid chip was then placed in the mixed QD EDC/NHS solution and incubated for 15 min at room temperature. During this incubation, the carboxyl functionalized QDs were bound to the microtoroid surface through the formation of an amide bond. After incubation, the chip was thoroughly rinsed with MES buffer, phosphate-buffered saline (PBS) buffer, deionized water, and ethanol to remove any unreacted reagents or residues. Finally, the chip was dried using nitrogen.

### Fitting

Spot intensity histograms were analyzed using a Gaussian Mixture Model (GMM)^76,77^ which is a parametric probability density function that combines multiple Gaussian component densities with different weights. The histogram was fitted with various numbers of Gaussian components in the GMM using the Expectation-Maximization algorithm^78,79^. To evaluate the fitting performance, the mean value of each peak was examined. It is expected that the mean values of the peaks are the product of the order number and the first peak mean value. After evaluation, it was determined that a GMM with four Gaussian components provided the best fit for the histogram in Fig. 4c. For the mixed QDs histogram in Fig. 4f, the same methodology is applied, resulting in the identification of five Gaussian distributions.

### High sensitivity fluorescence image

A N-STORM 5.0 system was used with a CFI HP Apochromat 100X AC TIRF 1.49 NA objective (Nikon) and a 20 mW 405 nm laser unit (LU-NV, Nikon). Following the application of an AT-Qdot 800 filter set (Chroma), the fluorescence signal was captured using a back-illuminated EMCCD (electron-multiplying charge-coupled device) camera (iXon Ultra 897; Andor). The microtoroid chip was imaged in a dry state, positioned upside down on a MatTek dish with a coverslip bottom, and maintained at a room temperature of 22 °C. At least 1900 images were acquired to generate the video.

### Supplementary information

Supplemental Material
