# Supplementary material for: Single 5-nm quantum dot detection via microtoroid optical resonator photothermal microscopy
Source: Light Sci Appl. 2024 Aug 19;13:195. doi: 10.1038/s41377-024-01536-9 (PMC11333578; doi:10.1038/s41377-024-01536-9)
Supplement: Supplementary file 1 — Supplemental Material [file 41377_2024_1536_MOESM1_ESM.pdf]

Supplementary Information for:

Single 5-nm quantum dot detection via microtoroid optical resonator photothermal microscopy

Shuang Hao
Wyant College of Optical Sciences, University of Arizona
Tucson, Arizona, 85721, United States

Sartanee Suebka
Wyant College of Optical Sciences, University of Arizona
Tucson, Arizona, 85721, United States

Judith Su
Wyant College of Optical Sciences and Department of Biomedical Engineering
University of Arizona, Tucson, Arizona, 85721, United States

Address correspondence to; judy@optics.arizona.edu

1. Configuration of FLOWER based photothermal microscopy

Our photothermal microscopy system is constructed based on an optical sensing system known as FLOWER (frequency locked optical whispering evanescent resonator)[1]. FLOWER tracks the resonance shift of an optical microcavity using frequency locking. Fig. 1a shows a schematic of FLOWER based photothermal microscopy. A tunable external cavity diode laser (New Focus TLB-6712-P Velocity™) is employed as the probe laser. The probe laser output is connected to a lithium niobate phase modulator (IXblue NIR-MPX800-LN-0.1), which is driven by a 25 MHz oscillation dither signal. A polarization controller (PC) and a 50:50 fiber beam splitter (BS) are utilized to manipulate the phase-modulated probe laser. The PC is responsible for controlling the polarization of the probe laser to maximize the coupling efficiency between the microtoroid and tapered fiber. The BS splits the light into signal and reference arms, which are input separately into a high bandwidth balanced photodetector (New Focus Model 1807). In the signal arm, an optical fiber is thermally tapered using a hydrogen torch to achieve a diameter of approximately 1 μm, ensuring enhanced coupling efficiency[2–4]. The microtoroid optical resonator is evanescently coupled to the system through the tapered signal arm fiber. The output signal from the balanced receiver is multiplied by the dither signal and then time-averaged, resulting in the generation of an error signal as depicted in Fig. 2b. This error signal is proportional to the wavelength detuning between the laser and the microtoroid resonance. To control the laser wavelength, a proportional-integral-derivative (PID) controller is used. The PID controller receives the error signal and provides feedback to the probe laser controller to bring the absolute value of the error signal toward zero. Consequently, the probe laser wavelength is locked precisely at the whispering gallery mode (WGM) resonance wavelength of the microtoroid. By monitoring the output of the PID controller, the resonance wavelength shifts can be accurately measured.

Based on the FLOWER system, a continuous wave (CW) laser (Thorlabs S3FC405) is utilized as the pump laser for particle excitation. To generate a high photothermal signal, a 405 nm wavelength is chosen as the excitation wavelength, as many molecules and particles exhibit characteristic absorptions in the UV spectrum. The pump laser is subjected to amplitude modulation (AM) using the input signal derived from the function generator. The output of the fiber-coupled pump laser is converted into a free-space beam using a fiber collimator. This collimated pump beam is directed toward a mirror that is mounted on the galvo driver. As the galvo mirror rotates, the angle of incidence of the collimated beam varies at the back focal plane of the relay lenses. The reflected beam then passes through relay lenses,

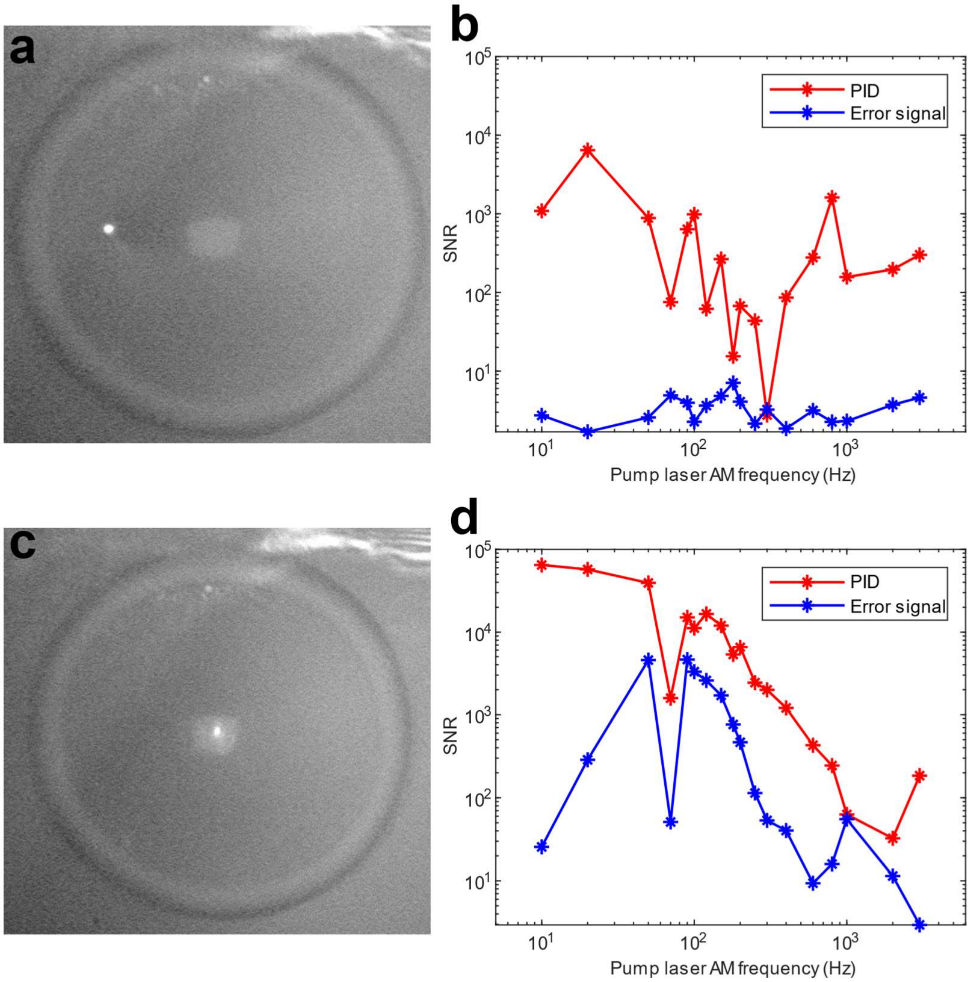

Fig. S1. Signal-to-noise ratio (SNR) of the photothermal signal. **a**, The pump laser spot is focused on the microtoroid disk area. **b**, Comparison of the SNR between the two photothermal signal sources (either from the PID or from the error signal) for a small photothermal response (at the disk). **c**, The pump laser spot is focused on the microtoroid pillar. **d**, Comparison of the SNR between the two photothermal signal sources for a large photothermal response (at the pillar). In **b** and **d**, PID output signal is input to the lock-in amplifier (red curve). The error signal is input to the lock-in amplifier (blue curve). The time constant is set at 1 s in the lock-in amplifier.

comprising a scan lens (f = 100 mm) and a tube lens (f = 100 mm). Subsequently, the beam is guided into a 60X objective lens via a cubic beam splitter. The free-space pump laser is focused onto the top surface of the microtoroid. The magnitude of the oscillation in the resonance shift, referred to as the

photothermal signal, is directly linked to the heat dissipation resulting from the pump laser. To accurately measure the photothermal signal, a lock-in amplifier (Stanford Research Model SR830) is employed, operating at the frequency of the AM. By utilizing this double lock-in technique, an enhanced signal-to-noise ratio (SNR) of the photothermal image is achieved through a 2D scan of the pump laser.

To mitigate potential damage to the integrated fiber-coupled laser caused by response delays in the constant power control loop, we avoid square wave or TTL modulation applied to the pump laser. Instead, sinusoidal wave AM is employed in the modulation input of the laser. The chosen AM frequency is set at 203.7 Hz. The selection of the modulation frequency requires a careful balance between three objectives: minimizing noise, maximizing the photothermal response of the microresonators, and minimizing the photothermal response time. Low-frequency technical noise, specifically 1/f noise, tends to increase at lower frequencies. By gradually increasing the modulation frequency, the spectral density of 1/f noise can be reduced. However, the re-etched microtoroid exhibits a limited bandwidth of 400 Hz for AM in the photothermal heating signal[5]. The photothermal response time decreases as the AM frequency increases. Taking all these factors into consideration, a reasonable range for choosing the AM signal falls between 200 Hz and 300 Hz. This range strikes a balance between minimizing noise, maximizing photothermal response, and optimizing the photothermal response time.

In our photothermal microscopy configuration, we have two viable options as the signal source for the lock-in amplifier: the PID output signal, used as feedback for tuning the probe laser, and the error signal, which is linearly proportional to the detuning of the microtoroid resonance from the probe laser wavelength. Fig. S1 provides a comparison of the SNR for these two photothermal signal sources. The R output of the SR830 lock-in amplifier serves as the photothermal signal, which represents the amplitude of the oscillating resonance shift signal at the AM frequency. In this context, SNR is defined as the ratio of the power of the photothermal signal to the power of the background noise.

The microtoroid structure consists of a silica disk supported by a small silicon pillar. When the pump laser scans the bare microtoroid's disk area, the photothermal signal is low due to the low optical absorption of the glass. However, when the pump laser scans the microtoroid pillar, the photothermal signal is high due to the large heat dissipation from the silicon pillar. This leads to a low background signal on the disk and a high background signal on the pillar. Therefore, the effective detection area is the microtoroid disk excluding the pillar.

In Fig. S1b, for small photothermal responses, the SNR of the PID photothermal response is generally

higher than that of the error signal photothermal response, except at an AM frequency of 180 Hz. This decrease in SNR at 180 Hz may be attributed to increased AM noise in the pump laser caused by issues with the laser control circuit. The SNR of the error signal remains below 10 at 180 Hz, resulting in a less noticeable decrease in SNR. A similar abnormal dip in SNR is observed at 70 Hz in Fig. S1d, for both the PID and error signal photothermal responses. In Fig. S1d, for large photothermal responses, the SNR of the PID photothermal response is also higher than that of the error signal photothermal response. Choosing an AM frequency of 203.7 Hz strikes a balance between photothermal SNR and response time. At this frequency, both for large and small photothermal responses, the PID method exhibits a 10 times higher SNR compared to the error signal method. Therefore, the PID photothermal response is more suitable for the re-etched microtoroid.

The re-etched microtoroid, with its small pillar, provides better thermal isolation, resulting in reduced heat dissipation conducted through the pillar to the chip substrate. This improved thermal isolation enhances the sensitivity of the photothermal response. However, it also decreases the microtoroid's AM cutoff bandwidth from 4 or 5 kHz to 400 Hz[5]. When the pump laser operates at low-frequency AM, the probe laser effectively tracks the microtoroid resonance, resulting in a consistently low detuning between their wavelengths. Consequently, the error signal exhibits low sensitivity to the photothermal response. Conversely, the PID output signal is highly sensitive to the photothermal response because it controls the probe laser wavelength. Therefore, for the re-etched microtoroid, the PID photothermal response is a better choice than the error signal method.

2. Cross-section absorption of Au nanosphere

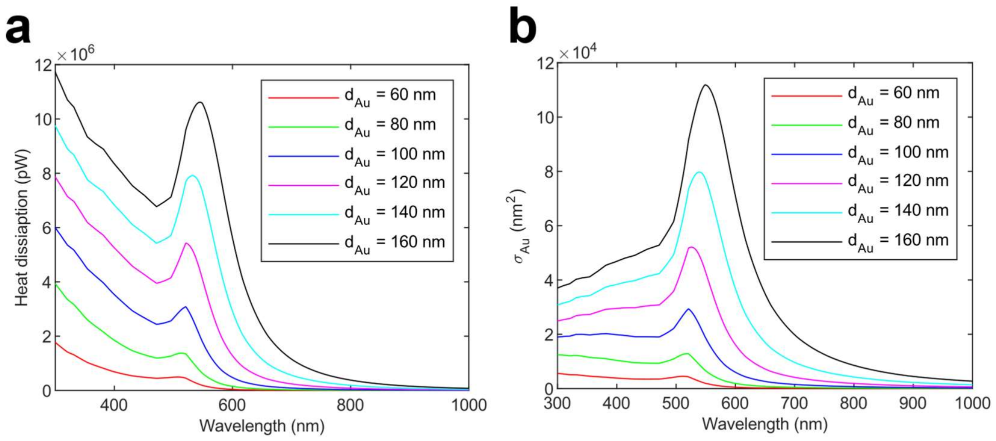

Fig. S2. Calculated optical and thermal properties of Au nanosphere. **a**, The absorption cross section of a gold (Au) nanosphere with diameter ranging from 60 nm to 160 nm. At 405 nm, the absorption cross section of a 100 nm Au nanosphere is $\sigma_{\mathrm{Au}} = 1.97 \times 10^4\ \mathrm{nm}^2$. **b**, The heat dissipation of the gold nanosphere in **a**. The heat dissipation of the 100 nm Au nanosphere mentioned above under a 405 nm pump laser $P_{\mathrm{heat}} = 3.43 \times 10^{-6}\ \mathrm{W} = 3.43 \times 10^6\ \mathrm{pW}$.

The absorption cross section σ_{Au} of individual Au nanoparticles is determined based on the Mie theory absorption model[6],

$$\sigma_{\mathrm{Au}} = \frac{2\pi}{\lambda} Im\{\alpha\} \tag{1}$$

where λ is the wavelength of illuminating light, the corrected polarizability α is[7]

$$\alpha = 3V \frac{1 - (\varepsilon + \epsilon_m)\,\theta^2/10}{\frac{\varepsilon + 2\epsilon_m}{\varepsilon - \epsilon_m} - \frac{(\varepsilon + 10\epsilon_m)\theta^2}{10} - i\,\frac{2{\epsilon_m}^{3/2}\theta^3}{3}} \tag{2}$$

where $\theta = \frac{2\pi r_{\mathrm{Au}}}{\lambda}$ is the size parameter and r_{Au} is the Au nanosphere radius. The relative permittivity ϵ_m is approximately 1 in air. The relative permittivity of Au nanosphere, ε, is taken from Johnson and Christy's report[8].

Fig. S2a shows the absorption cross section σ_{Au} of a gold nanosphere. The diameter of the nanospheres ranges from 60 nm to 160 nm. Notably, the absorption cross section exhibits a peak at around 520 nm. As the size of the Au nanospheres increases, the absorption cross section σ_{Au}

consistently demonstrates an upward trend. In our experiments we used a 100 nm Au nanosphere as our first test particle. The absorption cross section of 100 nm Au nanosphere is $\sigma_{\mathrm{Au}} = 1.97 \times 10^4\ \mathrm{nm}^2$ at 405 nm. These absorption cross section results provide insight into the optical properties of the Au nanosphere.

To calculate the heat dissipation of the Au nanosphere, it is essential to determine the intensity of the pump laser spot. The diameter of the pump laser can be calculated using the Rayleigh criterion, which states that:

$$d_{\mathrm{spot}} = 1.22 \times \frac{\lambda}{NA} \tag{3}$$

where the pump laser wavelength $\lambda = 405\ \mathrm{nm}$, and NA is the numerical aperture of the pump light illumination system. The spot intensity is:

$$I_0 = \frac{P_0}{S_{\mathrm{spot}}} = \frac{4P_0}{\pi d_{\mathrm{spot}}{}^2}, \tag{4}$$

where P_0 is the power of the laser spot focused on the microtoroid top surface. The 405 nm pump laser spot intensity is $17.4\ KW \cdot \mathrm{cm}^{-2}$. The target particle absorbed power equals to the heat dissipation of the Au nanosphere,

$$P_{\mathrm{heat}} = P_{\mathrm{abs}} = I_0\, \sigma_{\mathrm{Au}} \tag{5}$$

The heat dissipation of the Au nanosphere is calculated based on its absorption cross section, as shown in Fig. S2a, using Eq. (5). The heat dissipation of the Au nanosphere under 405 nm pump laser excitation is illustrated in Fig. S2b. Specifically, for a 100 nm Au nanosphere, the heat dissipation is calculated to be $P_{\mathrm{heat}} = 3.43 \times 10^{-6}\ \mathrm{W} = 3.43 \times 10^{6}\ \mathrm{pW}$ under a 405 nm pump laser. In previous reports, the microtoroid has the capability to detect heat dissipation power of a few tens of pW[9] and in this work a heat dissipation power of 0.75 pW. Therefore, the 100 nm Au nanosphere exhibits a significant photothermal response making it easily detectable.

3. Characterizations of QDs

When considering luminescent particles as the target, the absorption calculation presents a different scenario, as a portion of the absorbed light energy is transferred to emitted light. In this case, the fraction of heat dissipation in fluorescence particles is:

$$\eta_{\mathrm{heat}} = 1 - \eta_{\mathrm{fl}} + \frac{\eta_{\mathrm{fl}}\left(\frac{1}{\lambda_{\mathrm{exc}}} - \frac{1}{\lambda_{\mathrm{fl}}}\right)}{\frac{1}{\lambda_{\mathrm{exc}}}} = 1 - \eta_{\mathrm{fl}} \frac{\lambda_{\mathrm{exc}}}{\lambda_{\mathrm{fl}}}, \tag{6}$$

where η_{fl} is the quantum yield of the fluorescence particle, λ_{exc} is the excitation wavelength, and λ_{fl}

is the emission wavelength. The absorbance of the fluorescence particle solution was measured using a Nanodrop. According to the Beer-Lambert law, the molar extinction coefficient $\varepsilon_{\mathrm{ext}}$ is calculated in units of $M^{-1}cm^{-1}$,

$$\varepsilon_{\mathrm{ext}} = \frac{A}{cl}, \tag{7}$$

where A is the absorbance of the fluorescence particle solution, c is the molar concentration of the absorbing particles, l is the path length of the sample solution in unit cm. The cross-section absorption σ_{abs} is in unit cm^2, and can be calculated by,

$$\sigma_{\mathrm{abs}} = \varepsilon_{\mathrm{ext}} \times 10^{-1} \times In(10)/N_{\mathrm{A}} \tag{8}$$

in units of nm^2, where Avogadro's constant, $N_{\mathrm{A}} = 6.02 \times 10^{23}$. Finally, the heat dissipation is,

$$P_{\mathrm{heat}} = P_{\mathrm{abs}} \times \eta_{\mathrm{heat}} \tag{9}$$

The Qdot 800 QDs, supplied by Thermo Fisher Scientific have a diameter ranging from 18 nm to 20 nm. According to the certificate of analysis, these QDs have a quantum yield of 62% and emit light at a wavelength of 793 nm. On the other hand, the quantum yield of DiagNano 800 QDs, supplied by CD Bioparticles, was not provided, so it was determined by comparing the emission intensity of DiagNano 800 with that of Qdot 800,

$$\boldsymbol{\eta}_2 = \frac{I_2}{\varepsilon_2} \times \frac{\varepsilon_1}{I_1} \times \boldsymbol{\eta}_1, \tag{10}$$

where $\boldsymbol{\eta}$ is the quantum yield, ε is the molar extinction, I is the fluorescence emission intensity, with subscripts 1 and 2 representing DiagNano 800 and Qdot 800, respectively. The fluorescence emission intensities of the QDs were measured in the fluorescence image. The quantum yield of DiagNano 800 QDs was calculated to be 15.6%. These nanoparticles have a diameter ranging from 5 nm to 6 nm and exhibit similar fluorescence properties to Qdot 800, emitting light at a wavelength of 800 nm. The characteristic information of the two QDs used in the photothermal map is presented in Table 1. The heat dissipation of Qdot 800 is 363.1 pW, while the heat dissipation of DiagNano 800 is 71.3 pW. The ratio of their heat dissipation is approximately 5.1. In the experiment, the photothermal response ratio between the two QDs is approximately 5.4. The photothermal response is proportional to the heat dissipation. Therefore, the photothermal response ratio should remain consistent with the heat dissipation ratio.

4. WGM resonance shift COMSOL simulation

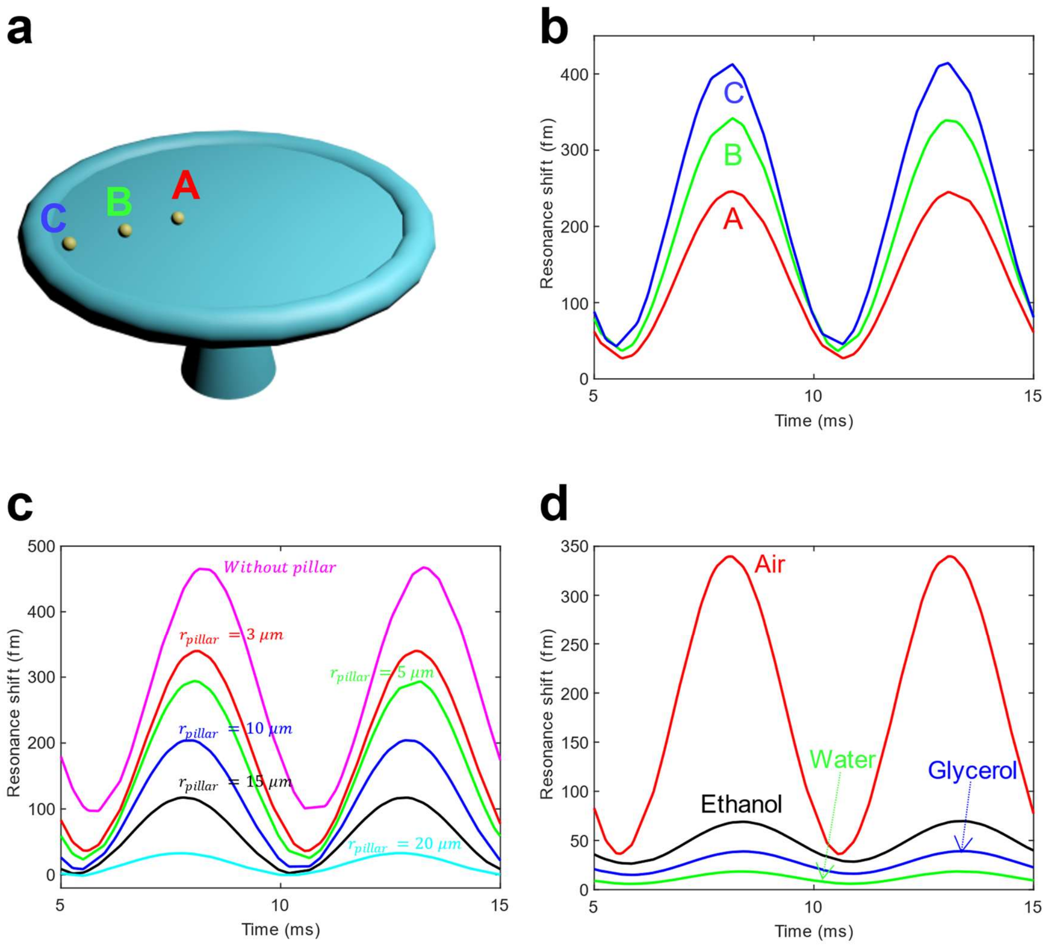

Fig. S3 COMSOL simulation of the microtoroid resonance shift. **a**, The rendered model of the microtoroid with 100 nm Au nanospheres positioned on the microtoroid resonator. The microtoroid resonator has a major diameter of 70 μm and a minor diameter of 3 μm supported by a silicon pillar ($r_{\mathrm{pillar}} = 3\ \mu\mathrm{m}$). The Au nanospheres A, B, and C are individually placed at distances of 10 μm, 20 μm, and 30 μm from the center of the microtoroid disk. **b**, The observed resonance shift resulting from the absorption of the 405 nm pump laser by the Au nanospheres A, B, and C in **a**. **c**, The resonance shift for different sizes of the supporting pillar. **d**, The resonance shift varies depending on the microtoroid's surrounding environment. In **c** and **d**, the Au nanosphere is placed at position B on the microtoroid supported by a pillar with radius of 3 μm.

The photothermal response is characterized by an oscillating resonance shift signal resulting from the absorption of the AM pump laser by the target particle. In the COMSOL simulation, the 100 nm Au nanoparticles serve as the heating source, with previously calculated heat dissipation of 3.43×10^{6} pW using a 405 nm pump laser. The pump laser spot size is larger than the size of the target particles. As a result, a significant portion of the light illuminates the top surface of the microtoroid. Since silica material exhibits minimal absorption, the light absorbed by the silica microtoroid can be neglected compared to the absorbed light by the particles. Most of the pump light is absorbed by the nontransparent silicon chip substrate, which is significantly larger in size compared to the microtoroid (with a major diameter of approximately 100 μm). Most of the heat dissipation in the substrate readily conducts throughout the entire chip substrate. However, due to the small diameter (below 5 μm) of the microtoroid's silicon pillar, the contribution of heat dissipation from the chip substrate to the photothermal signal is minimal. The simulation employs the finite-element analysis method. In the simulation, the heat dissipation process of the Au nanoparticles is simplified as point heat sources. The resonance wavelength shift signal is determined by the resonance condition of the optical resonator, which is defined by the resonance condition of optical resonator,

$$\lambda_{\mathrm{res}} = \frac{OPL}{m} \tag{11}$$

where λ_{res} is the resonance wavelength, OPL is the optical path length (OPL), m is the mode number of WGM. The OPL is calculated by integrating the product of the geometric length of the optical path followed by the WGM and the refractive index of the medium. The time-dependent simulation considers both heat transfer and thermal expansion simultaneously. The microtoroid's deformation caused by the thermal expansion from the point heat source is utilized for the calculation of the geometric length. The thermo-optic coefficient (dn/dT) of fused silica at room temperature (295 K) is $8.6 \times 10^{-6}\ \mathrm{K}^{-1}$. The refractive index information is acquired by the temperature distribution on the microtoroid’s rim. The resulting resonance shift of the 100 nm Au nanosphere at different positions on the microtoroid is illustrated in Fig. S3b. In the microtoroid's disk area, the resonance shift is larger when the particle is positioned closer to the microtoroid’s rim. By confining the photothermal spot selection to the annular region between points B and C, the variation in photothermal responsivity remains relatively consistent, with an approximate 20% margin of error. Fig. S3c depicts the simulated resonance shift with varying pillar size. The size of the pillar influences the thermal isolation of the microtoroid, with a smaller pillar

yielding better isolation. When the microtoroid absorbs the same optical energy from the pump laser, the enhanced thermal isolation leads to a higher temperature increase in the microtoroid. Consequently, a smaller pillar size enhances the microtoroid's photothermal sensitivity. In the case where the microtoroid lacks a pillar, the pillar is removed in the COMSOL model. Although this condition cannot be achieved in practice as the microtoroid would fall off without the pillar, it serves as an extreme hypothetical scenario to demonstrate maximum sensitivity. In Fig. S3d, the microtoroid is placed in different environments. The photothermal sensitivity in a glycerol environment is only 7.6% compared to that in ambient air. Thus, in glycerol, we estimate that a light absorption of 9.92 pW can be measured. In real wet environment experiments, additional challenges arise, such as the need for a chamber to hold the microtoroid in liquid, which increases the working distance of the objective lens and requires a decrease in numerical aperture, resulting in decreased resolution.

5. Photothermal spot of single QDs

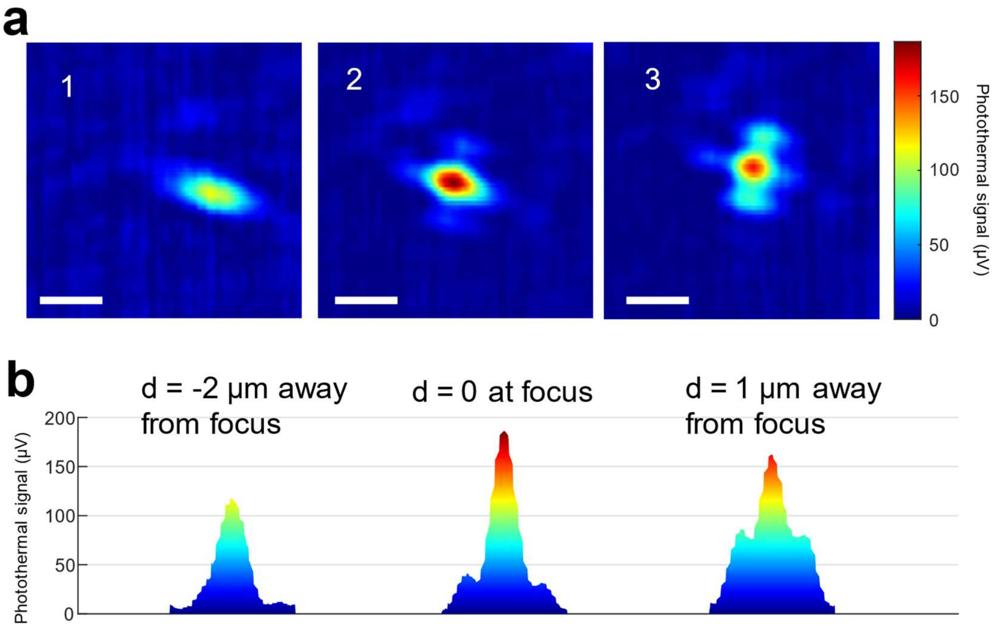

Fig. S4 Photothermal mapping of a single 5-6 nm QD at different focus positions.

a, Photothermal images of the same DN 800 QD (size: 5-6 nm) with the background signal removed. 1, The QD is positioned at d = -2 μm, out of focus and behind the focal point of the objective lens. 2, The QD is d = 0 precisely at the focus point. 3, The QD is located at d = 1 μm, appearing blurred in front of the focal point. Scale bar, 1 μm. **b**, Photothermal signal profiles and scans of the same DN 800 QD at varying distances from the focal point, corresponding to the photothermal maps 1-3 in **a**.

For the analysis of photothermal spot intensities of individual quantum dots, the Gaussian Mixture Model (GMM) is used to determine the photothermal spot peak distribution. This enables an assessment of the photothermal intensity distribution of the single quantum dots. Subsequently, a specific quantum dot is located and selected for a high-resolution scan (x-direction: 75 nm/pixel, y-direction: 9.4 nm/pixel). The difference in pixel resolution between the x and y directions arises from the microtoroid's 2D scanning procedure, which is carried out line by line along the y direction. Although the data acquisition card efficiently acquires data at a high sample rate for the y direction, the x direction is constrained by the number of scan lines. Enhancing resolution in the x-direction would substantially extend the scan time. Consequently, during the scan, the pixel resolution in the y-direction is designed to be higher than

that in the x-direction due to the sample rate of the lock-in amplifier output signal.

The results of photothermal mapping of single QDs at different focus positions are depicted in Fig. S4a. This photothermal map undergoes background signal subtraction, effectively yielding a final photothermal map with the original photothermal signal and estimated background removed. Such subtraction enhances the visibility of significant features within the data by minimizing the influence of background noise Firstly, we create a disk-shaped structuring element, often referred to as a kernel or mask, to define a local neighborhood around each pixel in the dataset. Next, we employ the "imopen" function to execute morphological opening on this disk-shaped structuring element. Morphological opening, a mathematical morphology operation, comprises two steps: erosion followed by dilation. During erosion, the structuring element is centered over each pixel in the input data, leading to the reduction of small bright spots and an overall decrease in image intensity. Then, dilation is applied with the structuring element centered over each pixel. This step enlarges the remaining features, emphasizing large structures. As a result of these two data processing steps, we acquire the background signal. Finally, by subtracting this background dataset, photothermal maps without the background are shown in Fig. S4a. This enables us to focus on the specific feature spots of interest, namely the photothermal spots of single QDs.

To directly illustrate the influence of focus conditions on photothermal spot intensity, Fig. S4b displays the profiles of the spots 1-3 with varying focus positions from Fig. S4a. In the initial scan, it's common for the quantum dot to be off the focus plane. As seen in Fig. S4a, the photothermal map of the first scan is depicted in Fig. S4a1, where the QD is positioned out of focus and behind the focal point. Here, the photothermal peak value is measured at 117.8 μV. With subsequent scans and focal plane adjustments, the photothermal map at the focus plane is presented in Fig. S4a2, showing photothermal peaks at 186.3 μV. In contrast, Fig. S4a3 portrays the quantum dot at d = 1 μm, resulting in a blurred appearance in front of the focal point. Consequently, the photothermal peak value diminishes to 162.2±1.7 μV. The photothermal signal, which is the lock-in amplifier output in voltage, measures the amplitude of the oscillating resonance shift. The SNR of the photothermal spot is defined as the ratio of the power of this oscillating voltage signal at the photothermal spot's peak to the power of the oscillating voltage signal in the surrounding background noise. The SNR of the photothermal spot for the 5 nm DN 800 QD reaches 1.18×10^{4}. The resulting noise floor from the photothermal map is measured at 1.69 μV.

6. High sensitivity fluorescence image of QDs

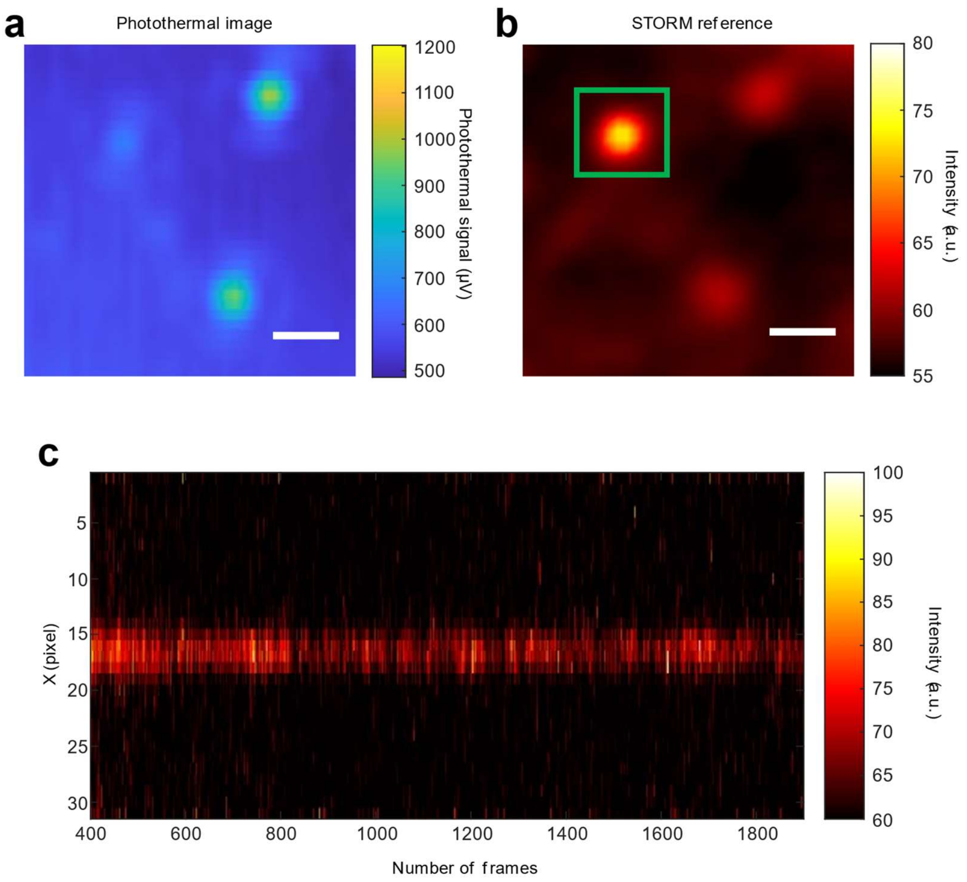

Fig. S5 Photothermal image of single QDs. a, Photothermal scan of Qdot 800 QDs (size:18-20 nm). **b**, Corresponding high sensitivity fluorescence reference image of **a**. **c**, The photo blinking behavior of single QDs within the marked green square in **b**.

To validate the accuracy of the photothermal images of QDs, we used fluorescence images of the QDs as references for the photothermal map. In our experimental procedure, after functionalizing the QDs onto the microtoroid, we executed a photothermal scan of the microtoroid, yielding the photothermal image of Qdot 800 QDs as depicted in Fig. S5a. Subsequently, the microtoroids containing the QDs were imaged using high sensitivity fluorescence microscopy. This involved a CFI HP Apochromat 100X AC TIRF 1.49 NA objective with a 405 nm laser for excitation. The Qdot 800 QDs (size: 18~20 nm) were successfully observed as shown in Fig. S5b. Both images reveal identical positions of the three QDs spots. A one-minute video is recorded. Single step photon blinking was observed and demonstrated in Fig. S5c. However, due to the lower quantum yield and smaller size, the single DN 800 QDs (size: 5-6

nm) were not observable. In the case of DN 800 QDs bound to the microtoroid, the count of fluorescence spots was much lower compared to the spots in photothermal image. This difference can be attributed to the fact that the single DN 800 QDs can't emit sufficient light for detection. Although, there may exist some single DN 800 QDs that are observable with an extended exposure time, their emitted signals remain too weak to observe the blinking effect in a suitable exposure time.

Supplementary References

1. Su, J., Goldberg, A. F. & Stoltz, B. M. Label-free detection of single nanoparticles and biological molecules using microtoroid optical resonators. *Light Sci. Appl.* **5**, e16001–e16001 (2016).
2. Harun, S. W., Lim, K. S., Tio, C. K., Dimyati, K. & Ahmad, H. Theoretical analysis and fabrication of tapered fiber. *Optik* **124**, 538–543 (2013).
3. Qing, P. *et al.* A simple approach to fiber-based tunable microcavity with high coupling efficiency. *Appl. Phys. Lett.* **114**, 021106 (2019).
4. Monifi, F., Özdemir, S. K., Friedlein, J. & Yang, L. Encapsulation of a Fiber Taper Coupled Microtoroid Resonator in a Polymer Matrix. *IEEE Photonics Technol. Lett.* **25**, 1458–1461 (2013).
5. Heylman, K. D. & Goldsmith, R. H. Photothermal mapping and free-space laser tuning of toroidal optical microcavities. *Appl. Phys. Lett.* **103**, 211116 (2013).
6. Myroshnychenko, V. *et al.* Modelling the optical response of gold nanoparticles. *Chem. Soc. Rev.* **37**, 1792–1805 (2008).
7. Kuwata, H., Tamaru, H., Esumi, K. & Miyano, K. Resonant light scattering from metal nanoparticles: Practical analysis beyond Rayleigh approximation. *Appl. Phys. Lett.* **83**, 4625–4627 (2003).
8. Johnson, P. B. & Christy, R. W. Optical Constants of the Noble Metals. *Phys. Rev. B* **6**, 4370–4379 (1972).
9. Heylman, K. D. *et al.* Optical microresonators as single-particle absorption spectrometers. *Nat.*

Photonics **10**, 788–795 (2016).
